# Clinical audit of an artificial intelligence (AI) empowered smile simulation system: a prospective clinical trial

**DOI:** 10.1038/s41598-024-69314-6

**Published:** 2024-08-21

**Authors:** Samar M. Adel, Yashodhan M. Bichu, Srirengalakshmi Muthuswamy Pandian, Waddah Sabouni, Chandani Shah, Nikhillesh Vaiid

**Affiliations:** 1 Department of Orthodontics, Faculty of Dentistry, Alexandria University, Champollion Street, El Azarita, Alexandria, Egypt; 2 Orthodontics (DSATP), Nobel Biocare Oral Health Centre/ Faculty of Dentistry, University of British Columbia, Vancouver, Canada; 3 Department of Orthodontics, Saveetha Dental College, Saveetha Institute of Medical and Technical Sciences, Chennai, India; 4 Bandol rivage orthodontie, Sanary sur Mer, France; 5 Mumbai, India; 6 Present Address: Department of Orthodontics, Saveetha Dental College, Saveetha Insitute of Medical and Technical Sciences, Chennai, India

**Keywords:** Smile aesthetics, Invisalign, Invisalign SmileView, Artificial intelligence, Smile prediction, Biotechnology, Medical research

## Abstract

Smile aesthetics is an important factor to consider during orthodontic treatment planning. The aim of the present study is to assess the predictability of Invisalign SmileView for digital AI smile simulation in comparison to actual smile treatment outcomes, using various smile assessment parameters. A total of 24 adult subjects (12 females and 12 males; mean age 22 ± 5.2 years) who chose to be treated using Invisalign were prospectively recruited to have their pretreatment smiles captured using the Invisalign SmileView to simulate their new smiles before treatment was started. Patients were then treated using upper and lower Invisalign aligners with average treatment time of 18 ± 6 months. Full post-treatment records were obtained and full smile frame images of simulated smile and actual final smile of each subject were evaluated by an independent examiner using an objective assessment sheet. Ten smile variants were used to assess the characteristics of the full smile images. Significance level was set at P < 0.05. The ICC for the quantitative parameters showed that there was an overall excellent & good internal consistency (alpha value > 0.7 & > 0.9). The Independent t test was performed amongst the quantitative variables. The P value was not significant for all except maxillary inter canine width (P = 0.05), stating that for the five variables namely; philtrum height, commissure height, smile width, buccal corridor and smile index, actual mean values were similar to the simulation mean values. For the qualitative variables, the Kappa value ranged between 0.66 and − 0.75 which showed a substantial level of agreement between the examiners. Additionally, the Chi square test for the qualitative variables, revealed that the P value was found to be significant in all except lip line. This implies that only the lip line values are comparable. More optimal lip lines, straighter smile arcs and more ideal tooth display were achieved in actual post treatment results in comparison to the initially predicted smiles. Five quantitative smile assessment parameters i.e., philtrum height, commissure height, smile width, buccal corridor, and smile index, could be used as reliable predictors of smile simulation. Maxillary inter canine width cannot be considered to be a reliable parameter for smile simulation prediction. A single qualitative parameter, namely the lip line, can be used as a reliable predictor for smile simulation. Three qualitative parameters i.e., most posterior tooth display, smile arc, and amount of lower incisor exposure cannot be considered as reliable parameters for smile prediction.

*Trial Registration number and date*: NCT06123585, (09/11/2023)

## Introduction

Comprehensive smile analysis is an integral component of the diagnosis and treatment planning in orthodontics and aesthetic dentistry. Based on neurological control, a smile can broadly be divided into an involuntary (or spontaneous) one and a voluntary (or posed) smile. An involuntary smile is related to emotion, whereas the posed or social smile is an intentional smile that usually is unrelated to emotions^1,2^.

There are a number of parameters that constitute the natural smile of an individual. These include smile line, smile arc, smile width and index, lower lip curvature, labiodental relationship, teeth display, buccal corridor, and position of incisal edge. In addition, dental-facial midline, symmetry, and gingival display also play an important role in the aesthetic appraisal of smile; and all these factors must be considered while designing a smile makeover. Furthermore, the norms for these smile characteristics may differ in different populations, thus the ethnicity should also be taken into consideration as another variable^3–5^.

Tooth movements in different planes expected during Invisalign clear aligner therapy are simulated by the proprietary ClinCheck software of Align Technology and the accuracy of these predictions have been previously investigated^6–8^. However, enhanced “smile aesthetics” is amongst the primary reasons as to why patients seek orthodontic therapy, whether with multi-banded appliances or aligners^9,10^. Akin to tooth movement simulations, Artificial Intelligence (AI) empowered smile simulations, also constitute an integral part of contemporary digital dentistry, allowing effective patient communication with the possibility of visualization of the patient’s treatment end goals.

Invisalign recently launched a smile simulation tool- SmileView; using which, by simply uploading a selfie captured on their smartphones, users can witness an instant, AI-generated, digital simulation of the transformation of their smile that they could expect with Invisalign treatment^11^. This application not only offers patients a glimpse into their future smiles but also serves as an invaluable communication tool between orthodontists and prospective patients. The tool is primarily a patient motivation and communication tool; results obtained may vary, and the company itself has made no claims about the accuracy of this application. Patient satisfaction is considered to be a very important treatment consideration and smile simulations might be a perceived benefit as it has been showcased that patients tend to change their appliances frequently^12–14^.

However, from a scientific perspective, just like the accuracy of tooth movement simulations has been evaluated, smile simulations also need to be evaluated for the accuracy of their predictions, to ensure that treating clinicians and patients themselves are aware of the limitations of these applications for effective and realistic communications. Therefore, the aim of the present study was to evaluate the predictability of Invisalign SmileView for digital smile simulation in comparison to the actual post-treatment smile outcomes measured using various photographic smile assessment parameters. The null hypothesis was that there is no agreement between predicted smile simulations generated by Invisalign SmileView and the actual smile obtained at the end of Invisalign clear aligner treatment for the various parameters used for smile assessment.

## Materials and methods

### Study design

The present study was a prospective consecutive clinical trial following the reporting guidelines for clinical trials of artificial intelligence interventions: the SPIRIT-AI and CONSORT-AI guidelines^15^.

### Ethical approval

This research was conducted in accordance with the relevant guidelines and regulations, as stated in the Declaration of Helsinki. Ethical approval was obtained from the Institutional Review Board of Saveetha Institute of Medical and Technical Sciences (SIMATS) (**SRB/SDC/ORTHO-2119/23/019**). All patients were informed of the nature of the study and signed informed consents accordingly.

### Sampling and selection criteria

A total of 24 patients (12 females and 12 males) with mean age 22 ± 5.2 years, with mild to moderate malocclusions requiring treatment with clear aligner therapy were recruited between 2018 and 2020, from private orthodontic offices in Dubai, United Arab Emirates of Invisalign Diamond Plus Elite practitioners with more than 15 years of experience. All cases included were in the Invisalign Lite category. All cases were Class I non extraction patients that were matched for irregularity index, having only minimal crowding^16^.

The sample size was calculated based on a study evaluating the perception of smile esthetics by dental students^17^. A power of the study set at 90%, the required minimum number of participants per group was 10, with a total of 20 subjects needed for the study execution. The sample size was calculated using the formula provided by Zhou in 2012, considering a desired power of 90%, a type I error rate of 5%, a type II error rate of 20%, a minimum acceptable precision of 0.97%, and an expected reliability (*ρ* ) of 0.787. Based on these parameters, approximately 20 subjects were deemed enough to be recruited and analysed for the study at the endpoint follow up which were increased to 24 to account for any dropouts during the study^18^.

Subjects were examined and screened, with the following eligibility criteria being considered- healthy systemic condition with no chronic diseases, no previous orthodontic treatment, adequate oral hygiene, and a healthy periodontium. All subjects received oral hygiene instructions and prophylaxis at least 2 weeks before records were taken. Full pretreatment and post treatment intraoral and extraoral records were taken for all the enrolled patients. The image is taken by the clinician/trained staff using the “Invisalign Practice App” which has an AI simulated Photo Uploader feature ( the Invisalign SmileView). This app is available on both the IOS and the Android Platforms. The simulated smiles are created only on those standardized images taken on the App. Head orientation, smile orientation and face orientation marks are prompts that indicate to the clinician/photographer that the posturing is appropriate for image capture. For a given patient, this positioning is standardized as the algorithm uses facial markers to indicate smile posture for photographs. In addition, the patients were trained as a part of a standard protocol to rehearse a social smile before image acquisition was attempted. The AI based app also has adjustments for lighting.

Treatment was performed using upper and lower Invisalign aligners with an average treatment time of 18 ± 6 months. Images were saved in JPEG file format and saved with assigned code numbers to allow for blinded assessments. Adobe Photoshop CS6 (Adobe Systems, San Jose, CA, USA) was used to edit the full-smile images. Images were cropped to leave a proportionate area around the lips to eliminate the effect of other facial characteristics and skin color variations on the aesthetic evaluation. Images were finally converted to 5 × 3 inches, black and white, 2100 saturation, 300-dpi JPEG files, and inserted to Keynote (macOS Monterey, USA) for smile assessment^19^. Actual post-treatment facial photographs were captured using a Canon DSLR 90D fitted with a Canon Ring Lite Flash MR-14EX II and a Canon 60 mm EF-S F2.8 Macro USM lens, under standardized lighting conditions.

### Data collection for objective smile assessment^20,21^

Ten smile variables were assessed digitally on the full smile images and image tool for macOS Monterey was used for the measurements. The enlargement ratio of each image was calculated by comparing the true length from the scale and measured length in the corresponding scale framed image. An assessment worksheet was created and filled in after a detailed analysis of the frontal pictures of a natural smile of each patient posttreatment and the simulated smile picture pre-treatment. Images were displayed on a laptop computer (MacBook Pro, USA). A single independent examiner (SA) performed all assessments. A definition was given for each of the measured outcomes together with an illustrative image showing the different variants of each category and the measurements landmarks. The worksheet used for the analyses of the considered smiles is reported in Table 1. Each set of smile photographs (post-treatment actual and pre-treatment simulation) was evaluated and compared for the following parameters.

**Table 1:** Objective assessment of the ten smile variables.

Smile parameter	Definition	Representative figure
Smile line or lip line:	Defined as the extent of the vertical tooth display on smiling or the elevation of the upper lip in relation to the maxillary incisors. Three types of smile lines have been described- high, average, and low. A high smile shows the maxillary anterior teeth along with a significant amount of gingiva, an average smile line shows maxillary anterior teeth with only interproximal gingiva, and a low smile line typically shows less than two-thirds of the maxillary anterior teeth^20,22^	Figure 1
Smile arc:	Is the relationship between the curvature of the maxillary anterior teeth and upper border of the lower lip. It is defined by drawing a line along the maxillary central incisal edges to the cusp tips of maxillary canines, which is related to another line drawn across the superior border of the lower lip. In subjects whose maxillary teeth are covered by lower lip, smile arc is designated as "not available". Three types of smile arcs have been described- “parallel to the teeth” (when the two lines follow the same curvature) also called a consonant smile, and a non-consonant smile, if the two lines are not parallel. A non-consonant smile can either be “straight” (with flatter curvature of the maxillary anterior teeth in relation to the lower lip) or “reverse” (when the maxillary anterior teeth form a reverse curve in relation to lower lip)^23^	Figure 2
The most posterior teeth displayed:	Smiles are categorized as displaying teeth up to the canines, first premolars, second premolars, or the first molars. A tooth is counted when more than half of its surface is visible^24^	Figure 3
The philtrum height	Is measured in millimeters from subspinale (the base of the nose at the midline) to the most inferior portion of the upper lip on the vermilion tip beneath the philtral columns^25,26^	Figure 4
Commissure height	Is measured in millimeters from a line constructed from the alar bases through subspinale and then from the commissures perpendicular to this line. The differential lip growth exhibits the difference in height in philtrum and commissural height in adolescents^26^	Figure 5
Smile width/Inter-commissure width	Is the distance from outer commissure to outer commissure on smile in millimeters^27–29^	Figure 6
Smile index	(inter commissural or smile width/inter-labial gap), is the ratio between the horizontal smile width and the vertical smile height during smiling (smile width/smile height)^26^	Figure 7
Maxillary inter-canine width	Defined as the distance from the distal aspect of the right canine to the distal aspect of the left canine in millimeters^29^	Figure 8
Buccal corridor	Is the space created between the buccal surface of the posterior teeth and the lip corners when the patient smiles. Orthodontists often refer to buccal corridors as “negative spaces”. The buccal corridor is measured from the mesial line angle of the maxillary first premolars to the interior portion of the commissure of the lips. Moore et al.^30^. have previously quantified a buccal corridor of 28% as medium-narrow, 15% as medium, 10% as medium-broad, and 2% as broad smile fullness^31^	Figure 9
Amount of lower incisor show:	Can be categorized into either 1) No show of lower incisors during smiling, 2) partial lower incisor exposure or 3) full length lower incisor exposure^32^	Figure 10

Figs. 11 and 12 demonstrate actual cases analyzed in the study, comparing the actual posttreatment smiles to the simulated pretreatment smiles.

Calibration was carried out on 7 cases before conducting the study. The measurements were repeatedly conducted until an acceptable level of agreement is achieved. All the measurements were performed by a single blinded operator (SA) using a digital caliper. The same measurements were then repeated for 7 cases 2 weeks later by the same operator (SA) and another independent operator (SP), to test for intra- and inter-operator reliabilities.

### Statistical analysis

All data was entered into a computer by assigning a coding system, proofed for entry errors. Data were entered in Microsoft Excel spreadsheet and analyzed using SPSS software (IBM SPSS Statistics, Version 20.0, Armonk, NY: IBM Corp.). The statistician was blinded to the nature and objectives of the study. Cronbach’s alpha and Intra Class Correlation Coefficient (ICC) were performed to check the internal consistency and agreement between two or more examiners or techniques (inter and intra) for the quantitative parameters- philtrum height, commissure height, smile width, maxillary inter-canine width, buccal corridor and smile index. The reliability or consistency strength of the questions used in the present study was determined using Cronbach's alpha correlation test and Cohen’s Kappa coefficient (κ) was used to measure the reliability in terms of probability of agreements versus probability of non-agreements for the qualitative parameters (such as most posterior tooth display, smile arc, amount of lower incisor show and lip line). Shapiro Wilks normality test was used to determine the normal distribution of data. Data was normally distributed, so parametric tests were employed. A P value of 0.05 was considered to be statistically significant. Quantitative outcome parameters were expressed in mean and standard deviation and qualitative outcome parameters were expressed in frequency and percentage. Independent t test was used to compare the quantitative outcome parameters between actual and simulation groups, and Chi square test was used to compare the qualitative outcome parameters between actual and simulation groups.

### Ethics approval and consent to participate

This study was performed in line with the principles of the Declaration of Helsinki. Ethical approval was obtained from the Institutional Review Board of Saveetha Institute of Medical and Technical Sciences (SIMATS) (**SRB/SDC/ORTHO-2119/23/019**). Informed consent was obtained from all individual participants included in the study.

### Clinical relevance

AI based Invisalign SmileView tool for pretreatment smile simulation can be used with limited predictability, based on the conditions of the present study.

**Figure 1:**
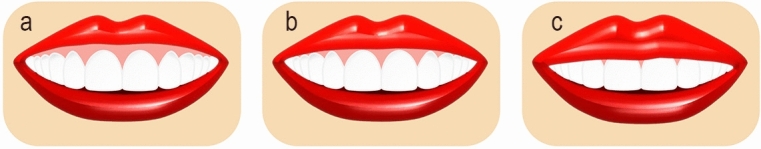
Illustrative diagram showing different lip lines.

**Figure 2:**
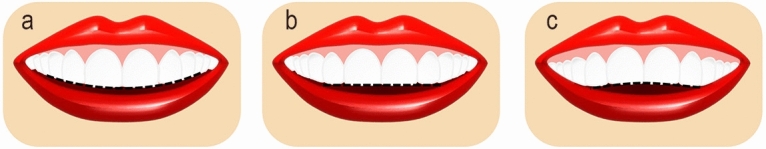
Illustrative diagram showing different smile arcs.

**Figure 3:**
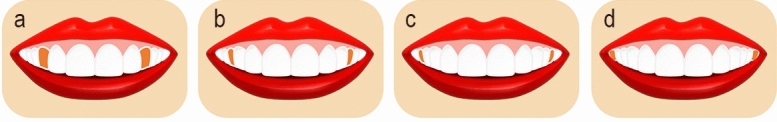
Illustrative diagram showing most posterior tooth display.

**Figure 4:**
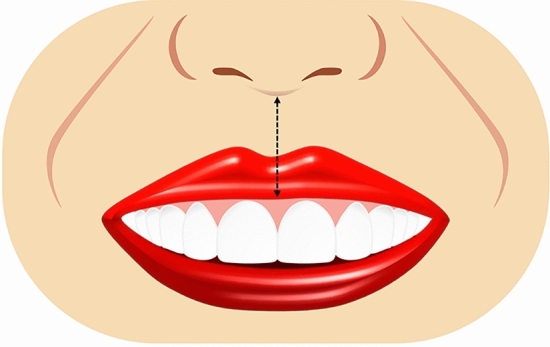
Illustrative diagram showing philtrum height in mm.

**Figure 5:**
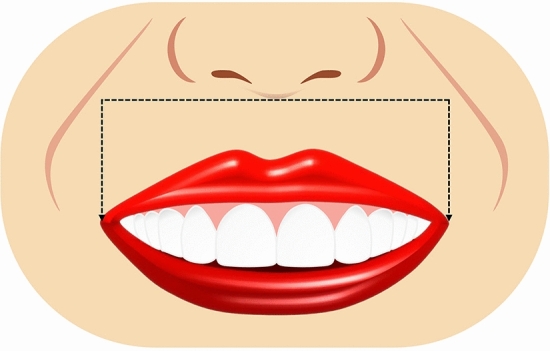
Illustrative diagram showing commissure height in mm.

**Figure 6:**
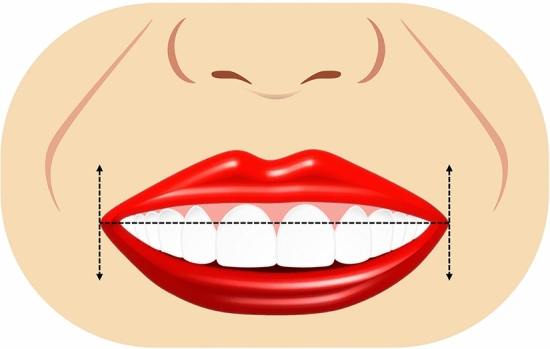
Illustrative diagram showing smile width in mm.

**Figure 7:**
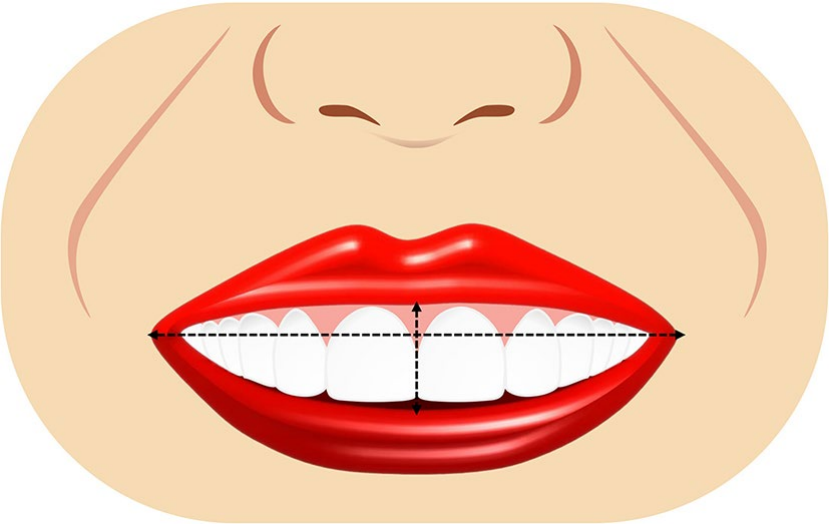
Illustrative diagram showing smile index in mm.

**Figure 8:**
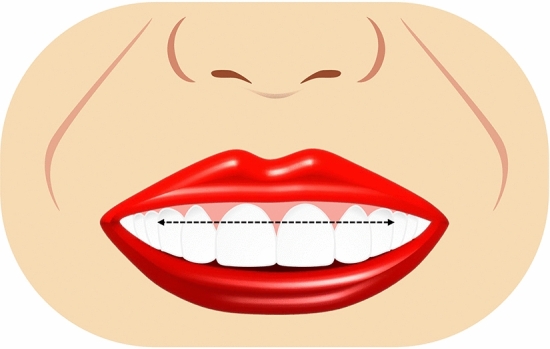
Illustrative diagram showing maxillary inter-canine width in mm.

**Figure 9:**
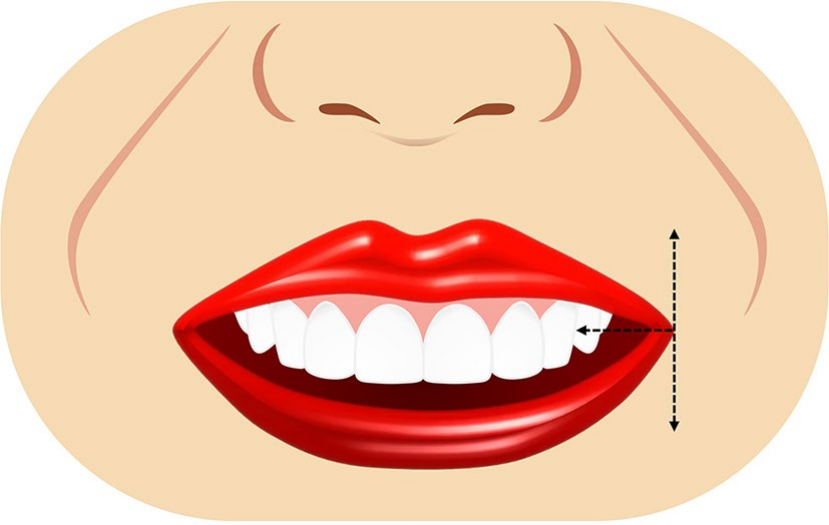
Illustrative diagram showing buccal corridor.

**Figure 10:**
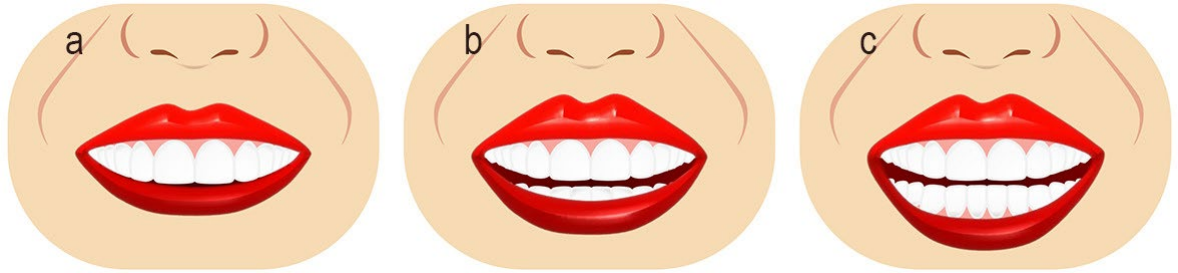
Illustrative diagram showing amount of lower incisor exposure.

## Results

In the present study, ten common smile variables affecting the beauty of a smile- six quantitative variables (philtrum height, commissure height, smile width, maxillary inter-canine width, buccal corridor, and smile index) and four descriptive variables (lower incisor show, lip line, smile arc and number of tooth display during smile) were studied. The actual post-treatment outcome obtained was compared with the simulated smile obtained with SmileView for these parameters.

**Figure 11:**
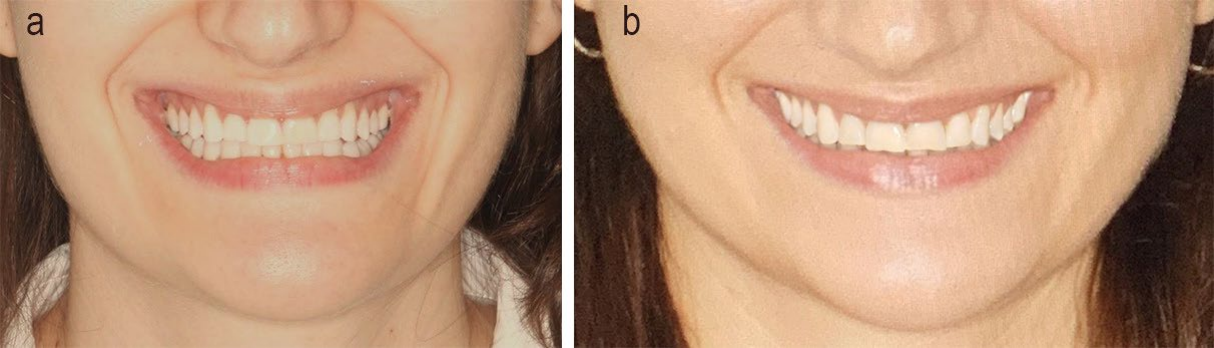
A female patient enrolled in the study showing the actual and simulated smiles.

**Figure 12:**
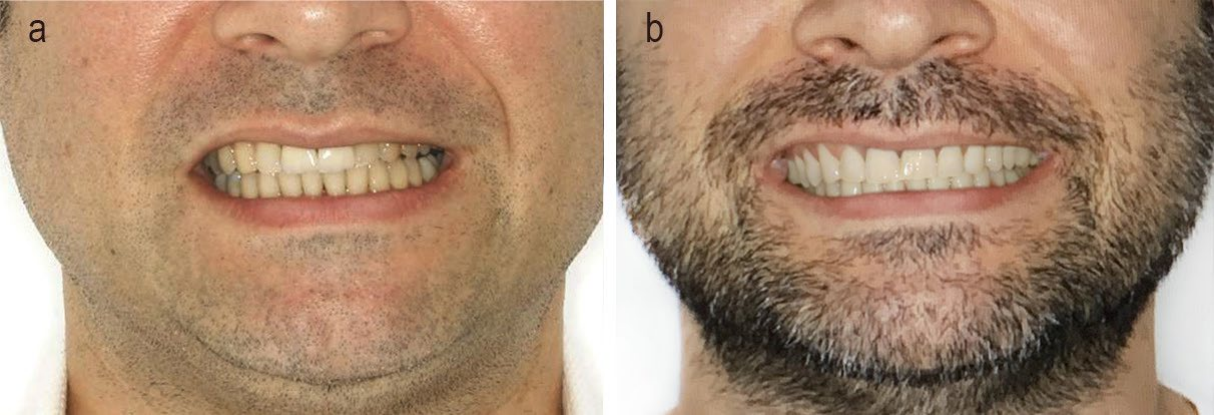
A male patient enrolled in the study showing the actual and simulated smiles.

Table 2. illustrates Intra Class Correlation (ICC) for quantitative parameters of smile assessment, while Table 3 illustrates the level of agreement for qualitative outcomes. The ICC for the quantitative parameters ranged between 0.7 and − 0.8 showing that there was an overall excellent & good internal consistency. The reliability or consistency strength of the questions used in the present study was determined using Cronbach's alpha correlation test. The Cronbach's alpha value ranged between 0.8 and 0.9 which denoted a good and acceptable level of reliability for the questions. The kappa value for the qualitative parameters ranged between 0.66 and − 0.75 for qualitative parameters which showed a substantial level of agreement between the examiners.

**Table 2:** Intra Class Correlation for quantitative parameters of smile assessment.

Variable	Cronbach's Alpha	IntraClass Correlation	Lower Bound	Upper Bound
Philtrum height (Actual) (Simulation)	.867	.764	.528	.891
Commissure height (Actual) (Simulation)	.899	.817	.623	.917
Smile width (Actual) (Simulation)	.938	.883	.750	.948
Maxillary inter-canine width (Actual) (Simulation)	.940	.886	.755	.949
Buccal corridor (Actual) (Simulation)	.862	.795	.623	.895
Smile index (Actual) (Simulation)	.876	.734	.558	.873

**Table 3:** Level of agreement for qualitative outcomes.

Measures of agreement	Most posterior tooth display	Lip line	Smile arc	Amount of lower incisor exposure
Kappa value	0.68	0.75	0.72	0.66

**Figure 13:**
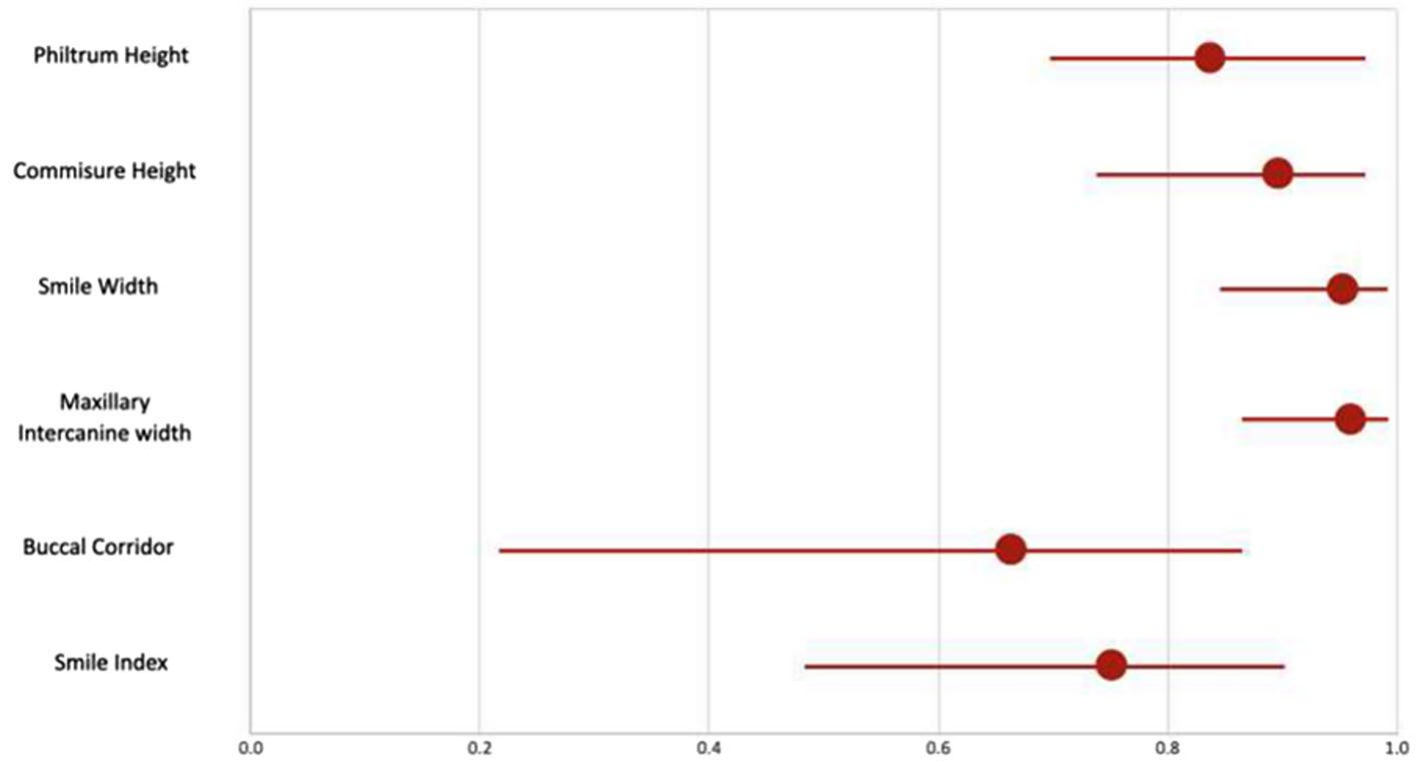
Diagrammatic representation of forest plot depicting agreement between the quantitative parameters of smile assessment.

**Figure 14:**
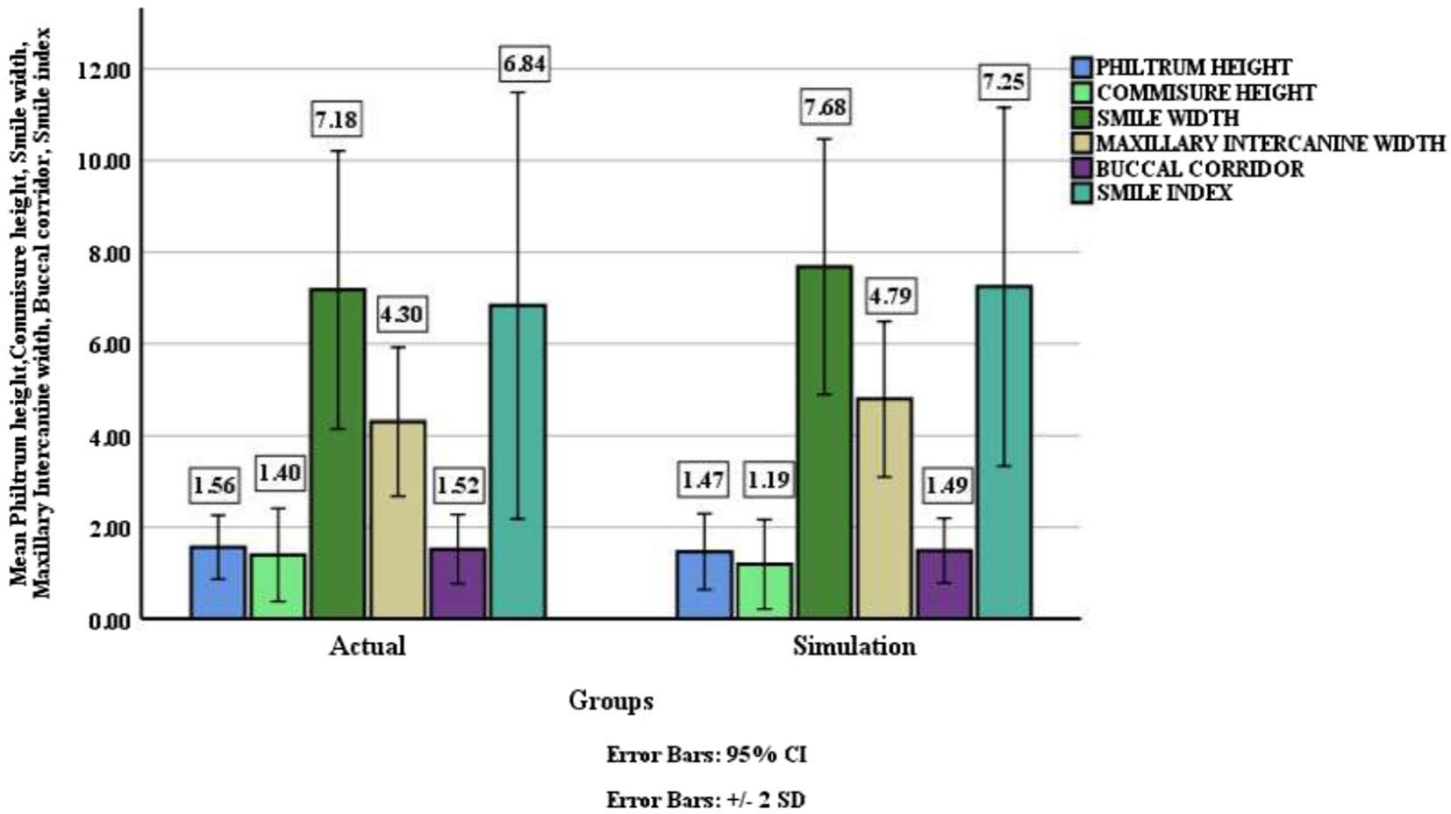
Mean values of the quantitative variables (Philtrum height, Commissure height, Smile width, Maxillary inter-canine width, Buccal corridor, and Smile index) recorded in the actual and simulation group.

Table 4 illustrates a comparison of the quantitative outcome parameters between actual and simulation groups using independent t test. On comparison of the quantitative variables, the results demonstrate that P value was not significant for all the variables except maxillary inter canine width, meaning that for the five variables namely; philtrum height, commissure height, smile width, buccal corridor and smile index, actual mean values were similar to the simulation mean values and hence could be used as predictors. The mean value for the philtrum height for the simulation group was 1.47 ± 0.41 which was comparable to the mean of the actual group 1.56 ± 0.35. (P > 0.05). This implies that there is no statistically significant difference between the two groups. Similarly, the mean values of commissure height, smile width, buccal corridor and smile index in the simulation group were comparable to the actual group with P > 0.1. This indicates that there is no statistically significant difference between the groups. Figure 13 shows a diagrammatic representation of forest plot depicting agreement between the quantitative parameters of smile assessment, and Fig. 14 demonstrates mean values of the quantitative variables (philtrum height, commissure height, smile width, maxillary inter-canine width, buccal corridor, and smile index) recorded in the actual and simulation group.

**Table 4:** Comparison of quantitative outcome parameters between actual and simulation groups using independent t test.

Outcome	Groups	N	Mean (mm)	Standard Deviation (mm)	Independent t test value	P value
Philtrum height	Actual	24	1.56	0.35	0.86	0.39
	Simulation	24	1.47	0.41		
Commissure height	Actual	24	1.40	0.51	1.41	0.16
	Simulation	24	1.19	0.49		
Smile width	Actual	24	7.18	1.52	− 1.19	0.24
	Simulation	24	7.68	1.40		
Maxillary inter canine width	Actual	24	4.30	0.81	− 2.05	0.05*
	Simulation	24	4.79	0.85		
Buccal corridor	Actual	24	1.52	0.38	0.30	0.76
	Simulation	24	1.49	0.35		
Smile index	Actual	24	6.84	2.33	− 0.66	0.51
	Simulation	24	7.25	1.96

**Figure 15:**
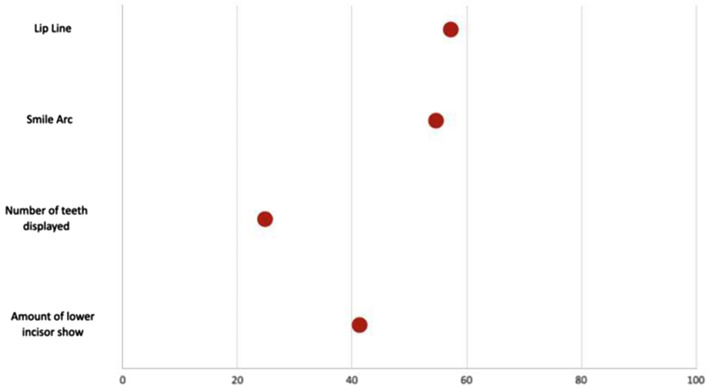
Diagrammatic representation depicting percentage accuracy for the qualitative parameters of smile assessment.

Amongst the four qualitative variables, the P value was found to be significant in all except lip line. This implies that only the lip line values are comparable and can be used as a predictor for simulation. Table 4 depicts that, with respect to the most posterior tooth display, 91.7% of actual treated group participants displayed up to first molars when compared to the simulation group which was only 33%. The P value was found to be statistically significant and accordingly, the parameter is considered not reliable for prediction. For the smile arc, 45.8% and 50% of the treated patients had straight and reverse smile arcs, while the simulated group had 4.2% and 95.8% respectively. Hence, the P value was found to be statistically significant, and the parameter cannot be used for prediction. For the amount of lower incisor exposure parameter, the treated group consisted of 54.2% showing partial lower incisor show and 45.8% showing full lower incisor show when compared to the simulated group which exhibited 4.2% and 95.8% respectively making the P value highly significant and implying that the parameter cannot be used for prediction. The lip line was the only parameter that showed comparable results with 16.7% showing average lip line and 79.2% showing low lip line and this was in close proximity to the simulated group which showed 16.7% and 83.3% respectively, thus it can be used a reliable parameter for prediction. Table 5 illustrates a comparison of the qualitative variables (most posterior tooth display, lip line, smile arc, and amount of lower incisor exposure) using Chi-square test and Fig. 15 shows a diagrammatic representation depicting percentage accuracy for the qualitative variables.

**Table 5:** Comparison of qualitative variables using Chi-square test.

Qualitative outcome parameter	Categories	Groups		Chi-square test value	P value
		Actual n (%)	Simulation n (%)		
Most posterior tooth display	Displaying up to first premolars	0(0%)	3(12.5%)	17.60	0.001**
	Displaying up to second premolars	2(8.3%)	13(54.2%)		
	Displaying up to first molars	22(91.7%)	8(33.3%)		
Lip line	High	1(4.2%)	0(0%)	1.02	0.59
	Average	4(16.7%)	4(16.7%)		
	Low	19(79.2%)	20(83.3%)		
Smile arc	Parallel to teeth	1(4.2%)	0(0%)	12.79	0.002**
	Straight	11(45.8%)	1(4.2%)		
	Reverse	12(50.0%)	23(95.8%)		
Amount of lower incisor exposure	No lower incisor show during smile	0(0%)	0(0%)	14.52	0.001**
	Partial lower incisor show during smile	13(54.2%)	1(4.2%)		
	Full lower incisor show during smile	11(45.8%)	23(95.8%)

## Discussion

Improvement in smile aesthetics is one of the major reasons why patients seek orthodontic treatment. By addressing smile aesthetic concerns, patients can experience improved self-confidence, enhanced social interactions, and increased satisfaction with their overall appearance. In the era of digital transformations, patients have one-touch access to various apps and online tools that can facilitate smile makeovers. These digital tools offer interactive and convenient means for patients to explore different smile enhancement options, visualize potential outcomes and communicate better with treating clinicians.

Bichu et al. in a scoping review in 2021 highlighted an exponential increase in the number of studies evaluating various applications of Artificial Intelligence (AI) and Machine Learning (ML) in the field of orthodontics over the past three decades, with the most commonly utilized AI domain focusing on various aspects of orthodontic diagnosis and treatment planning^33^. An increasing body of literature has focused on AI based apps and their impact on orthodontic care delivery in the current landscape^34–37^. Ryu and coworkers in 2022 built an automated system based on Convolutional Neural Network (CNN) model for the image type classification of clinical orthodontic photos, that included four facial and five intraoral photos. CNNs deep learning technique was employed to classify orthodontic clinical photos according to their orientations. This study demonstrated a 98.0% valid prediction rate for both facial and intraoral photo classification and suggested that AI can be applied to digital color photos to assist in the automation of the orthodontic diagnosis process^38^. Intra oral images have been assessed using CNN by Talaat et al. as well, exhibiting an accuracy of 99.99%, precision of 99.79% and a recall of 100%^39^.

Invisalign, the world’s leading clear aligner brand, recently launched a tool called Invisalign SmileView, to enable patients to visualize their post treatment smiles by an artificial intelligence-based simulation algorithm. Patients only need to upload their selfie photograph captured on a smartphone and the SmileView tool can simulate their smiles makeover in a few seconds. This tool is primarily a patient motivation and communication tool; the results obtained may vary, and the company itself has made no claims about the accuracy of this application.

Scholarly literature has studied in depth the predictability of clear aligners to achieve the desired tooth movement treatment outcomes by comparing the predicted movements with the final achieved ones^6–8^, improving our understanding of the capabilities and the limitations of clear aligner therapy. Moreover, these simulations allow patients to visualize different tooth movements needed in order to correct their malocclusions. The accuracy of digital model registrations to assess the predictability of movements has also been studied, revealing that different software algorithms can yield different accuracies^40–42^.

Whether the simulated smile is predictable in comparison to the actual smile achieved post treatment using Invisalign, still needs to be validated from a scientific perspective, akin to tooth movement simulations for the accuracy of their predictions, to ensure that treating clinicians and patients themselves are aware of the limitations of these applications. Therefore, the objective of the present prospective clinical trial was to assess the predictability of the Invisalign SmileView tool.

The reason why Lite patients were selected is essentially to standardize the quantum of change. Lite patients with just 14 aligners and a maximum of single refinement, will only be advocated for patients seeking minimal changes to anterior teeth only. Thus when the quantum of change with orthodontics is minimal, the simulation of the photographs should be more accurate. With more complex malocclusions, greater confounding variables can occur.

Multiple features of a smile can be assessed to comprehensively evaluate smile aesthetics and the present study evaluated ten common smile variables affecting the beauty of a smile- six quantitative variables (philtrum height, commissure height, smile width, maxillary inter-canine width, buccal corridor, and smile index) and four descriptive variables (lower incisor show, lip line, smile arc and number of tooth display during smile). The actual post-treatment outcome obtained was compared with the simulated smile obtained with SmileView tool for these parameters.

Ideally, the first step to be planned in aesthetic smile treatment is the vertical position of maxillary incisors which is represented by the smile arc^43^. An ideal smile arc has the incisal edges of maxillary teeth paralleling the contour of the lower lip. Other types of smile arc also exist, such as straight or reverse smile arcs^44^. The curved smile arc gives a more youthful appearance than the straight or reverse types. This highlights the need for individualized orthodontic bracket bonding in the aesthetic zone, starting with the end in mind to achieve optimal smile arc. In the present study, the smile arc analysis revealed that 45.8% and 50% of the treated patients had straight and reverse smile arc, while the simulated group had values of 4.2% and 95.8% respectively for the same parameters. The P value for this parameter was found to be statistically significant, and it can thus be inferred that this parameter cannot be used reliably for the smile prediction provided by the SmileView tool; which implies that clinicians have to be cautious when predicting smile arc analyzed from digital simulations, in the conditions of the present study.

When the number of teeth displayed during smiling are considered, it has been shown that in an average smile in young adults, the six maxillary teeth and the first or second premolars are typically displayed, with less percentage of people showing first molars^20^. In the present study, a tooth display up to the first molars was achieved in 91.7% of cases as compared to only 33.3% ideal display in simulated smiles. These results indicate that better teeth display post-treatment will most probably be achieved in comparison to the predicted simulated smiles. The P value for this parameter was found to be statistically significant and accordingly, this parameter is considered not reliable for prediction.

The smile line can be classified as high, medium, or low according to Tjan et al.^20^. The lip line is considered ideal when the upper lip reaches the gingival margin, displaying the total length of the maxillary central incisors, together with the interproximal gingivae. However, it has been recently reported that gingival exposure of less than 3 mm is still considered to be perfectly acceptable revealing a more youthful smile, with greater gingival exposure being considered as high lip line with a gummy smile. On the other hand, a low lip line with less than maximal incisor exposure is considered to be esthetically unpleasing as it reflects an older looking smile^4,5^. The amount of vertical exposure is dependent on multiple variables including lip length, lip elevation, vertical maxillary height, crown height, vertical dental height, as well as incisor inclination. All these factors must be examined and analyzed during smile line assessment. In the current study, the results demonstrate a fair agreement between actual and simulated lip lines. However, more optimal lip line was observed in actual treatment outcomes as compared to simulated smiles. Hence, it can be considered that better smile lines maybe be achieved clinically than those predicted by the SmileView tool. The P value was found to be non-significant for the lip line, implying that among all the qualitative parameters, only the lip line values are comparable and can be used as a predictor for simulation.

Gingival and maxillary incisor display during smiling is considered to be a youthful feature of smile esthetics. However, with age advancement, especially in men, more lower incisor exposure occurs with significant decrease in maxillary incisor exposure during smiling^32^. In the present study, with regards to the amount of lower incisor exposure parameter, the treated group consisted of 54.2% showing partial lower incisor show and 45.8% showing full lower incisor show when compared to the simulated group which exhibited 4.2% and 95.8% respectively making the P value highly significant and implying that the parameter cannot be used for prediction. The findings of this study thus demonstrated a minimal agreement in the amount of lower incisor display on smile between simulated and actual post-treatment smiles, leading to the reference that the amount of lower incisor exposure cannot be considered highly predictable from the simulation tool.

Buccal corridor, defined as the bilateral space between the buccal surface of visible maxillary posterior teeth and the lip commissure during smiling, is another feature that is considered during smile analysis. There are different variants of buccal corridors including wide, intermediate, and narrow or non-existent corridors. There is no clear consensus in the literature about the aesthetic influence of buccal corridors on smile aesthetics. While some studies show that the different types of buccal corridors do not affect the aesthetic perception of smile, others believe that it can have a significant impact on smile attractiveness^45,46^. However, it is believed that intermediate buccal corridors are more aesthetic than narrow or even wide buccal corridors. The findings of the present study reveal that buccal corridor showed poor internal consistency and only fair agreement when simulated smiles were compared to the actual achieved ones. Based on previous studies demonstrating little importance of buccal corridors to the overall smile attractiveness, buccal corridors may minimally affect the overall perception of smile attractiveness of patients utilizing this Smile Simulation tool.

Similarly, the smile index achieved after treatment did not show satisfactory results when compared to simulated smile results, demonstrating only a moderate agreement. However, smile index was found to be a predominant factor in perceiving smile as attractive, revealing a more youthful smile appearance with a lower smile index^21^.

To our knowledge, the present study is the first clinical trial to assess the predictability of the SmileView tool. The study design being prospective, helped eliminate the selection bias that is inevitably present in retrospective studies. The single examiner who performed all the measurements and the statistician were blinded during measurements and analysis, therefore eliminating assessment bias. Additionally, intra and inter-operator reliabilities were calculated, showing excellent agreements in measurements. One of the factors that must be considered is that the lower face view used in this study may have facilitated the detection of small differences between the simulated smiles and actual smiles achieved compared with a full-face perspective. The authors however would like to state that in the first place, there are no commercial claims about the accuracy of prediction of the Invisalign SmileView tool. The feature is essentially a patient motivational tool, and the use of such tools has been illustrated for the said purpose in literature^47^; however, its efficacy is still an objective of a future trial. The present study aimed to evaluate its accuracy so that clinicians themselves are aware of the limitations of digital simulation applications and take these into consideration during patient communications. A limitation of this study is that the cases evaluated were only Invisalign Lite cases with mild to moderately severe malocclusions and a cohort of more complex malocclusions would probably provide additional information.

## Conclusions

Considering the limitations associated with this study, the following conclusions can be drawn:

1. Invisalign SmileView tool for pretreatment smile simulation can be used with limited predictability, based on the conditions of the present study.
2. More optimal lip lines, straighter smile arcs and more ideal tooth display were achieved in actual post treatment results in comparison to the initially predicted smiles.
3. Five quantitative smile assessment parameters namely philtrum height, commissure height, smile width, buccal corridor, and smile index could be used as reliable predictors of smile simulation. The sixth variable, maxillary inter canine width, however, cannot be considered to be a reliable parameter for smile simulation prediction.
4. A single qualitative parameter, namely the lip line, can be used as a reliable predictor for smile simulation. The other three qualitative parameters, namely most posterior tooth display, smile arc, and amount of lower incisor exposure cannot be considered to be reliable parameters for smile prediction.

## Future studies

1. Multicenter assessments can be done on larger sample sizes on different types and severities of malocclusions to draw more generalized conclusions.
2. Smile characteristics for different age ranges can be compared using this tool.
3. A study evaluating the impact of this tool, on patient and operator confidence in treatment, or patient conversions would also provide useful information.
4. Comparison between planned and post treatment 3D digital models with the smile simulation.

## Data Availability

All data generated or analysed during this study are included in this published article in the form of tables and figures.
